# Association between genetic risk score and tri-ponderal mass index growth trajectories among different dietary consumption adolescents in a prospective Taiwanese cohort

**DOI:** 10.1186/s12986-022-00718-9

**Published:** 2022-12-19

**Authors:** Yi-Fan Wu, Kuo-Liong Chien, Yang-Ching Chen

**Affiliations:** 1 Department of Family Medicine, Renai Branch, Taipei City Hospital, Taipei, Taiwan; 2 Institute of Epidemiology and Preventive Medicine, College of Public Health, National Taiwan University, Taipei, Taiwan; 3 Department of Psychology, National Chengchi University, Taipei, Taiwan; 4 Department of Internal Medicine, National Taiwan University Hospital, Taipei, Taiwan; 5 Department of Family Medicine, Taipei Medical University Hospital, Taipei, Taiwan; 6 Department of Family Medicine, School of Medicine, College of Medicine, Taipei Medical University, Taipei, Taiwan; 7 School of Nutrition and Health Sciences, College of Nutrition, Taipei Medical University, Taipei, Taiwan; 8 Graduate Institute of Metabolism and Obesity Sciences, Taipei Medical University, Taipei, Taiwan

**Keywords:** Single-nucleotide polymorphism, Tri-ponderal mass index, Adolescent growth trajectories, Gene-diet interaction

## Abstract

**Background:**

Single-nucleotide polymorphisms (SNPs) in various genetic loci are associated with childhood obesity; however, their influence on adolescent growth patterns has rarely been explored. This study investigated whether genetic variants could predict tri-ponderal mass index (TMI)-derived growth trajectories and the interaction between genetic and dietary factors.

**Methods:**

We conducted Taiwan Puberty Longitudinal Study, a prospective cohort that recruited 1,135 children since 2018. Anthropometric measurements were recorded every three months, while dietary nutrition assessment and biological sampling for genotyping were collected during the first visit. TMI growth trajectory groups were identified using growth mixture modeling. A multinomial logistic regression model for different growth trajectories was used to examine the effect of candidate SNPs, and the most related SNPs were used to establish the genetic risk score. We then explored the effect of the genetic risk score in subgroup analysis according to dietary calories and different dietary consumption patterns.

**Results:**

Three TMI-based growth trajectory groups were identified among adolescents. The “increased weight” trajectory group accounted for approximately 9.7% of the participants. *FTO*/rs7206790 was associated with the increased weight growth trajectory after adjusting for the baseline TMI and other correlated covariates (OR: 2.13, 95% CI: 1.08–4.21). We generated the genetic risk score using 4 SNPs (*FTO*/rs7206790, *ADCY9*/rs2531995, *TFAP2B*/rs4715210, and *TMEM18*/rs6548238) and selected the threshold of 10 points to define risk categories. There were 11.66% and 3.24% of participants belonged to the increased weight trajectory in high- and low-risk groups, respectively; and the predictive ability of the genetic risk score was notable among low calories intake participants (OR: 1.90, 95% CI: 1.18–3.05 vs. OR: 1.17, 95% CI: 0.78–1.75 in high calories intake group).

**Conclusion:**

Our results offer a new perspective on the genetic and dietary basis of changes in adolescent obesity status. Individualized interventions for obesity prevention may be considered among high-risk children.

## Introduction

Childhood obesity is a major public health concern with increasing global prevalence. It is associated with many noncommunicable diseases, such as type II diabetes, hypertension, dyslipidemia, and cardiovascular disease [1, 2]. However, some studies have found that individuals who are obese in adulthood but had a below-average weight during childhood have the highest risk of some cardiovascular diseases [3, 4]. Because adolescence is associated with rapid body development, obesity growth trajectories during this period may play a pivotal role in increasing the risk of cardiometabolic dysfunction [5, 6].

Body mass index (BMI) is a simple and common index for defining overweight and obesity in adults. However, the use of this index in adolescents and children is less convenient because the growth chart for the same sex and age is required as a reference. 7 The tri-ponderal mass index (TMI), which is defined as the weight (kg)/height (m^3^), was proposed as a better indicator of adolescent body fat composition [8]. It is useful for measuring overweight, and obesity in adolescents in clinical practice, and a persistent increase in the TMI growth trajectory during adolescence can predict diabetes in early adulthood more accurately than can BMI [9].

Obesity is believed to result from the interplay of the environment and innate genetic factors [10, 11]. Similar to obesity, several environmental factors are associated with the growth trajectory in childhood, including parental education level [12], a decrease in moderate to vigorous physical activity [13], and early puberty [14]. However, these factors may not completely explain the different growth patterns in adolescents. Researchers have identified more than 1,000 possible obesity-related loci [15] after the publication of the first obesity-related genome-wide association study (GWAS) in 2007, which demonstrated BMI-associated single-nucleotide polymorphisms (SNPs) in the *FTO* gene [16]. Nevertheless, no GWAS study has been conducted to explore the association between SNPs and TMI growth trajectories during adolescence.

We conducted a prospective longitudinal study to construct TMI growth trajectories among adolescent participants and then investigated the association between 17 obesity-related genetic polymorphisms and the different trajectory groups. Studies have shown that genetic influences on obesity may be modified by dietary fat intake [17, 18]; therefore, we explored whether the effect of genetic variants is altered by the participants’ different proportions of calories and main nutrient intake.

## Materials and methods

### Study population

The Taiwan Puberty Longitudinal Study (TPLS) is a multidisciplinary, longitudinal project. This project recruited children from pubertal and pediatric endocrine clinics of several hospitals [Taipei Medical University Hospital, Cathay General Hospital (CGH), Taipei Municipal Wanfang Hospital, and National Cheng Kung University Hospital (NCKUH)] beginning in 2018. Girls aged 6–14 years and boys aged 9–17 years were invited to participate and received prospective follow-ups. The exclusion criteria were (1) the presence of hereditary diseases, such as Turner syndrome and Prader–Willi syndrome; (2) the presence of neurological and psychiatric diseases, such as Tourette syndrome or attention-deficit/hyperactivity disorder; and (3) intake of medicines that may affect natural growth during adolescence, such as leuplin (leuprorelin acetate), oral steroids, and ritalin (methylphenidate). Parents or guardians had to complete an informed consent form at the baseline visit. This study was approved by the Institutional Review Board of Taipei Medical University (N201802018), that of CGH (CGH-P108107), and that of NCKUH (B-BR-108-076) and complied with the principles outlined in the Helsinki Declaration.

### Weight index measurement and other covariates

The participants’ weight and height were measured using an electronic scale, with shoes taken off, light clothing worn, and participants fasted beforehand. TMI was calculated as weight (kg)/height (m^3^), and the same measurement was performed at the first visit and then repeated every 3 months. Each participant’s parents or guardians were asked to answer a baseline characteristics questionnaire along with their children, which included questions related to the participants’ birth date, gender, postnatal information (birth weight and breastfeeding condition), family socioeconomic status (family income and parental education level), and environmental factors (home tobacco smoke exposure and incense burning). Moreover, a Chinese version of the International Physical Activity Questionnaire was used to calculate the average exercise time for all children [19].

At the first visit, a pediatric endocrinologist assessed the pubic hair and breast development of each girl and measured testicular volume using the Prader orchidometer for each boy [20]. If the two breasts of a girl were in different developmental stages or the testes of a boy were not identical, the stage of the more advanced side was adopted. Finally, pubertal development was then graded according to the 5-stage scale described by Tanner [21, 22].

### Nutrient intake assessment

Twenty-four-hour dietary recall was conducted by trained registered dietitians on two different days after the first recruitment. Energy and nutrient intakes were estimated on the basis of Nutritionist Edition, COFIT Pro, Version 1.0.0, a software package for nutrient analysis that features a Taiwanese food composition table as the nutrient database (Taipei, Taiwan). A validation study of COFIT has been published by our team [23]. The dietary records were coded and linked to this system to calculate nutrient and energy intake.

### Candidate SNP genotyping

Participants were requested not to eat or drink for at least 1 h before scraping the brush inside the mouth 10 times. The collection brush was then maintained at − 80 °C and transferred for DNA purification [24]. We applied the DNA extraction protocol using the Gentra Puregene Buccal Cell Kit 140 [24].

Genotyping was performed using the Sequenom MassARRAY iPLEX platform [25] at the National Center for Genome Medicine, Taiwan. In total, 17 candidate obesity-related SNPs identified from our previous unpublished study in a Taiwanese children cohort were selected, and most of them have also been reported to be associated with obesity from literature review (rs574367 [26], rs9356744 [26], rs12597579 [26], rs6567160 [26], rs3817334 [26], rs1555543 [26], rs4715210 [26], rs7206790 [27], rs6548238 [28], rs9939609 [29], rs1421085 [30], rs12463617 [31], rs8053360 [32], rs16858082 [33], rs7498665 [34], and rs4788102 [35]) except rs2531995. After polymerase chain reaction amplification, shrimp alkaline phosphatase incubation, primer extension, and chip dispensing, data acquisition and automated genotype calling were performed through matrix-assisted laser desorption/ionization time-of-flight mass spectrometry.

### Statistical analysis

The TMI growth trajectories were constructed with growth mixture modeling (GMM) by using Mplus software version 7. GMM was conducted to explore an individual’s pattern of weight change with latent trajectory classes. It allowed different trajectory groups to vary around different means and let each latent class have its estimates of variances and covariate influences [36]. The participants’ TMI from every trimonthly visit was adopted for growth trajectory identification. Using the full-information maximum likelihood method, missing values were managed in GMM by assuming them to be missing at random [37]. We tested the number of trajectories from two to five and examined the trajectory pattern as linear, quadratic, or cubic to find a suitable model. The Bayesian information criterion (BIC) and the bootstrap likelihood ratio test (BLRT) were used to determine the model’s adequacy. We ensured at least 5% of participants were sorted to each group.

For descriptive analyses, baseline participant characteristics in each growth trajectory group were compared using the chi-square test and analysis of variance for categorical and continuous variables, respectively. Multinomial logistic regression was used and odds ratios (ORs) of genetic variants for specific growth trajectories were reported after adjustment for age, sex, and baseline confounding covariates. Although the parents or guardians of some participants omitted the information concerning family income and education level, we still included these participants with missing data in our analysis using the missing indicators method [38].

To explore the combined effects of obesity-related SNPs on growth trajectory, an additive model was used to determine the genetic risk score (GRS). We selected risk alleles with a p of ≤ 0.15 in regression analysis and weighted them according to their coefficients in the regression model [39]. We then used logistic regression to evaluate the effect of the GRS and the predictive ability among different cut-off points.

For gene-diet interaction investigation, we used all participants’ average daily dietary calories and average percentage of calories from carbohydrate, fat and protein as the cut-off points to define different dietary consumption patterns. The same regression method was applied to explore whether different associations existed between the GRS and increased weight growth trajectory among high- or low-calories/nutrients intake subgroups. Statistical significance was defined by a two-tailed p of < 0.05. All analyses were performed using SAS version 9.3 (SAS Institute).

## Results

**Fig. 1:**
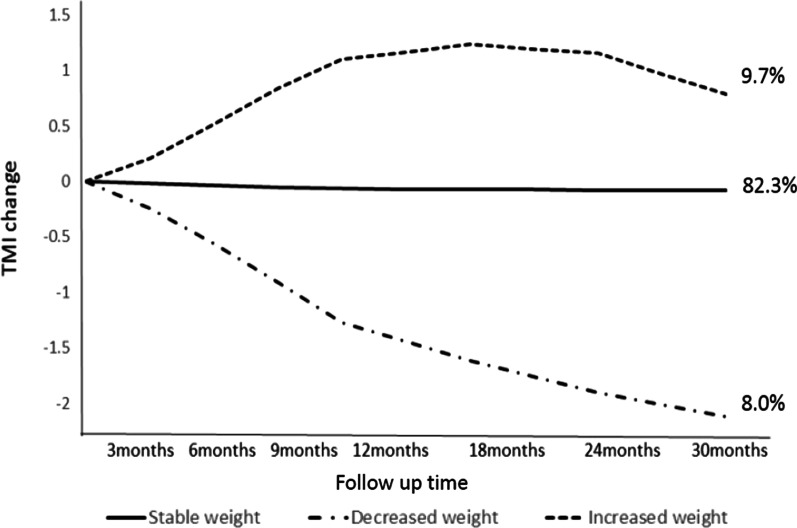
Three TMI-derived growth trajectory groups identified using growth mixture modeling

A total of 1,135 participants in the TPLS, for whom follow-up data for > 6 months were available, were used to establish growth trajectory groups (average follow-up: 4.4 times, 414 days). After excluding the model with < 5% participants in each trajectory and conducting an adequacy assessment by using the BIC and BLRT, we determined three TMI growth trajectory groups (Fig. 1). Among the three groups, most children were categorized as the “stable weight” group (n = 934, 82.3%) and demonstrated almost no change in the TMI during the follow-up period. The TMI decreased in approximately 8.0% (n = 91) of the participants, whereas it increased in the remaining 9.7% (n = 110) children during the observation period.

Table 1 presents the basic characteristics of each trajectory group. In the stable weight growth pattern group, the baseline TMI was significantly lower and the daily calorie intake per kilogram of weight was higher than that in the other two groups. The participants in the “increased weight” group appeared to have a lower socioeconomic status, which is consistent with the results of a previous study [12]. Concerning pubertal development, the probability of the early Tanner stage was higher in the “decreased weight” group and lower in the increased weight group.

**Table 1:** Basic characteristics of three TMI-based growth trajectory groups in the Taiwan Puberty Longitudinal Study

	All participants	Stable weight	Decreased weight	Increased weight	*p*-value^c^
Participants number	1135	934	91	110	
Age	10.4 (2.0)	10.4 (1.9)	10.7 (1.8)	10.2 (2.1)	0.25
Girls	750 (66.3)	637 (68.4)	43 (47.8)	70 (64.2)	< 0.01
Baseline TMI, kg/m^3^	13.1 (2.6)	12.4 (2.1)	15.8 (1.8)	16.5 (2.6)	< 0.01
Birth weight, kg	3.0 (0.5)	3.0 (0.5)	3.1 (0.5)	2.9 (0.5)	0.09
Breast feeding ^a^	787 (81.6)	653 (82.6)	62 (80.5)	72 (74.2)	0.13
Parental education level ^a,b^					0.02
Senior high school or below	60 (6.3)	45 (5.8)	4 (5.3)	11 (11.7)	
College or University	530 (56.0)	423 (54.4)	50 (65.8)	57 (60.6)	
Institute	357 (37.7)	309 (39.8)	22 (29.0)	26 (27.7)	
Family income^a, b^					< 0.01
< 70,000 NTD per month	206 (22.0)	156 (20.2)	15 (20.3)	35 (37.6)	
70,000 ~ 150,000 NTD per month	465 (49.6)	384 (49.8)	39 (52.7)	42 (45.2)	
>150,000 NTD per month	267 (28.5)	231 (30.0)	20 (27.0)	16 (17.2)	
Tanner stage ^a^					0.04
Stage 1 or 2	696 (80.3)	582 (80.1)	65 (91.6)	49 (71.0)	
Stage 3 or 4	131 (15.1)	110 (15.1)	5 (5.0)	16 (23.2)	
Stage 5	40 (4.6)	35 (4.8)	1 (1.4)	4 (5.8)	
Exercise habit, > 30 min per day^a^	450 (59.1)	385 (61.0)	33 (50.0)	32 (50.0)	0.07
Daily calories, Kcal	1598.7 (425.8)	1595.0 (424.3)	1628.8 (422.2)	1604.5 (444.0)	0.86
Daily calories from carbohydrate, %	47.7 (9.6)	47.7 (9.3)	47.7 (10.3)	48.3 (11.2)	0.85
Daily calories from fat, %	33.8 (8.3)	33.9 (8.2)	33.5 (8.4)	33.1 (8.9)	0.76
Daily calories from protein, %	15.5 (3.2)	15.6 (3.1)	16.0 (3.7)	14.9 (3.5)	0.11

Numbers are present as mean (SD) or n (%)

*NTD* new Taiwan dollars, *TMI* Tri-ponderal mass index

^a^Information from some participants were unavailable due to missing data

^b^The sum of all categories’ percentage ≠ 100% due to rounding

^c^ANOVA test for continuous variables and Chi-square test for categorical variables between three growth trajectory groups

We then investigated the effect of the 17 candidate SNPs and found only 1 SNP (*FTO* rs7206790) to be significantly associated with the increased weight growth trajectory (adjusted OR: 2.13, 95% confidence interval (CI): 1.08–4.21, *p*-value: 0.03 for additive model; Table 2). Because there were missing information for some participants, for example, around 16.6% participants didn’t provide parental educational level, and the data of family income were not available in 17.4% participants (Table 1), we performed a sensitivity analysis that excluded all participants with missing variables. The adjusted OR was 2.46 for *FTO* rs7206790 for increase weight growth trajectory, though the *p*-value was not siginificant due to insufficient power (*p*-value: 0.54, total participants number: 743). For GRS construction, we selected 4 genes with p ≤ 0.15 in regression analysis to generate the GRS (*FTO*/ rs7206790, *ADCY9*/ rs2531995, *TFAP2B*/ rs4715210, and *TMEM18*/ rs6548238; Table 2). The number of risk alleles was counted for each SNP, and rs7206790 and rs6548238 were double-weighted according to the coefficients in regression analysis to generate a GRS ranging from 0 to 12. We defined GRS ≥ 10 as the threshold of the high risk group because of the best discrimination ability (Table 3), and 11.66% and 3.24% of participants belonged to the increased weight trajectory in the high- and low-risk groups, respectively (*p* < 0.001). In addition, we also used 5-fold cross-validation method to test the GRS, and the result is consistent.

**Table 2:** Association of 17 candidate SNP alleles with the increased weight growth trajectory in the Taiwan Puberty Longitudinal Study

SNP	Nearest gene	Chr.	Allele		Increased weight vs. Stable weight		
			Effect	Other	OR^a^	95% CI	*p* value
rs7206790	*FTO*	16	C	G	2.13*	1.08, 4.21	0.03
rs2531995	*ADCY9*	16	T	C	1.47	0.91, 2.37	0.11
rs4715210	*TFAP2B*	6	C	T	1.66	0.85, 3.28	0.14
rs6548238	*TMEM18*	2	C	T	1.95	0.78, 4.84	0.15
rs12463617	*TMEM18*	2	C	A	1.92	0.77, 4.80	0.16
rs1555543	*PTBP2*	1	C	A	1.73	0.77, 3.91	0.18
rs8053360	*IRX3*	16	C	T	1.98	0.58, 6.77	0.28
rs9939609	*FTO*	16	T	A	1.32	0.71, 2.46	0.37
rs7498665	*SH2B1*	16	A	G	1.15	0.62, 2.15	0.66
rs4788102	*SH2B1*	16	G	A	1.15	0.61, 2.15	0.67
rs12597579	*GP2*	16	C	T	1.09	0.68, 1.76	0.71
rs9356744	*CDKAL1*	6	T	C	1.07	0.71, 1.61	0.76
rs3817334	*MTCH2*	11	T	C	1.07	0.68, 1.67	0.80
rs574367	*SEC16B*	1	G	T	1.08	0.61,1.92	0.80
rs1421085	*FTO*	16	T	C	1.07	0.60, 1.90	0.82
rs6567160	*MC4R*	18	C	T	1.03	0.60, 1.75	0.92
rs16858082	*GNPDA2*	4	T	C	1.03	0.64, 1.65	0.92

*Chr.* chromosome, *CI* confidence interval, *OR* odds ratio, *SNP* single-nucleotide polymorphism, *TMI* Tri-ponderal mass index

^a^Multinomial logistic regression with the additive model for each SNP, adjusted for gender, age, baseline TMI, parental education level, family income, Tanner stage, and exercise habit

*p-value < 0.05

**Table 3:** The performance of different threshold of GRS to predict increased weight TMI growth trajectory

GRS	OR^a^	95% CI	*p*-value	Participants with Increased weight in different risk categories
8	2.02	0.61, 6.63	0.25	GRS ≧ 8 (90.4%) vs. GRS < 8 (9.6%): 8.39% vs. 4.41%
9	2.27*	1.01, 5.10	0.04	GRS ≧ 9 (77.0%) vs. GRS < 9 (23.0%): 9.12% vs. 4.29%
10	4.00*	1.99, 8.05	< 0.001	GRS ≧ 10 (56.6%) vs. GRS < 10 (43.4%): 11.66% vs. 3.24%
11	2.34*	1.35, 4.05	0.003	GRS ≧ 11 (28.1%) vs. GRS < 11 (71.9%): 13.00% vs. 6.05%

*CI* confidence interval, *GRS* genetic risk score, *OR* odds ratio

^a^Adjusted for gender and age

*p-value < 0.05

**Fig. 2:**
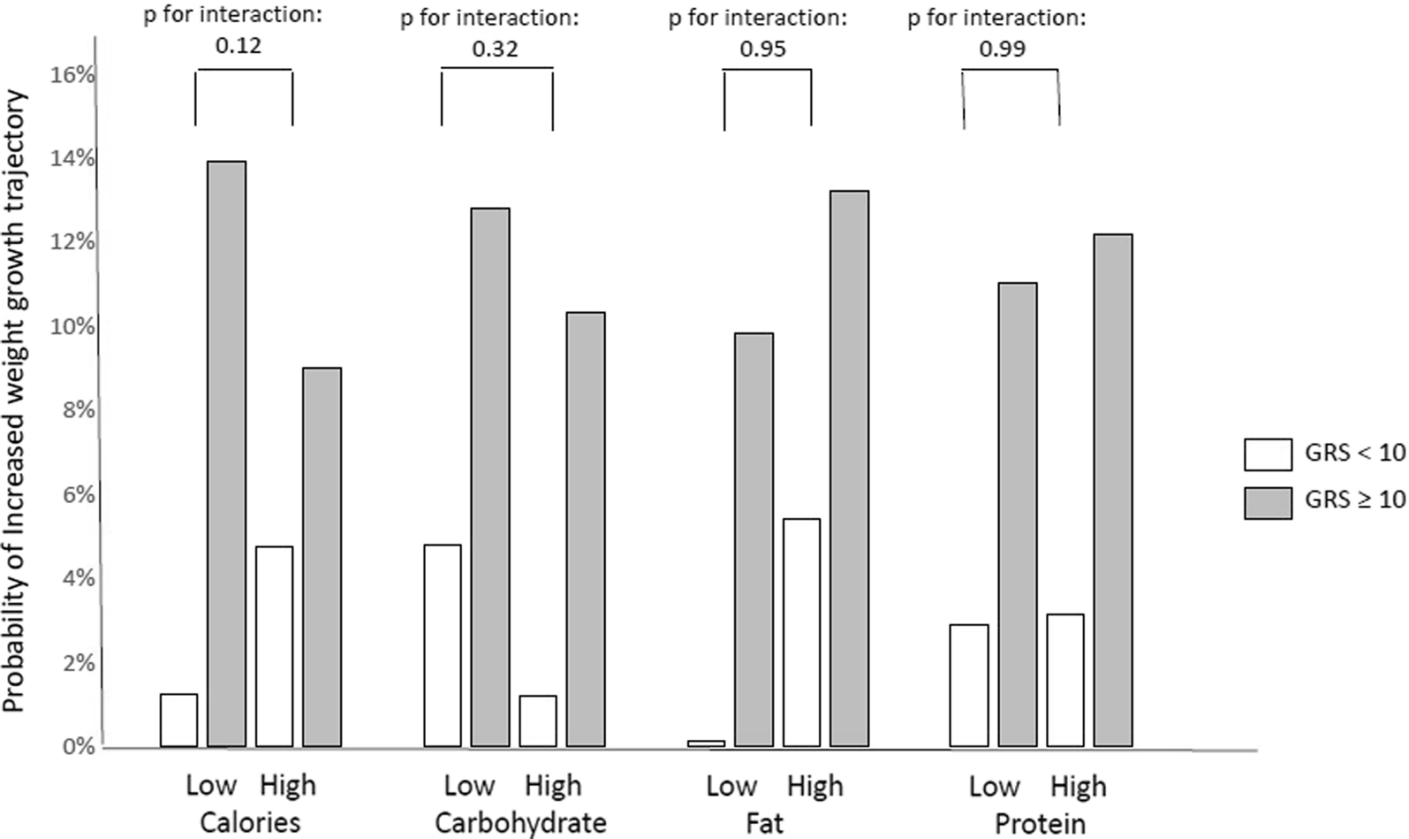
The performance of genetic risk score among different dietary consumption subgroups

For the dietary calories and nutrient intake subgroup analysis, the cut-off points for daily calories, carbohydrate, fat and protein intake were 1598.7 Kcal, 47.7%, 33.8% and 15.5%, respectively. Though no significant gene–diet interaction was found, our GRS exhibited relatively different effects between the paired subgroups. For instance, the GRS was associated with an increased risk of the increased weight trajectory in children consuming on a low-calories diet (OR: 1.90, 95% CI: 1.18–3.05, *p*-value: 0.01), but the influence on children with high-calories intake was not significant (OR: 1.17, 95% CI: 0.78–1.75, *p*-value: 0.44; Table 4; Fig. 2).

**Table 4:** Odds ratios of GRS for increased weight TMI growth trajectory in different nutrients intake subgroups

	OR^a^	95% CI	*p*-value	*p* for interaction
All participants	1.46*	1.17, 1.82	< 0.001	
High calories intake	1.17	0.78, 1.75	0.44	0.10
Low calories intake	1.90*	1.18, 3.05	0.01	
High carbohydrate intake	1.71*	1.03, 2.84	0.04	0.46
Low carbohydrate intake	1.38	0.95, 2.02	0.09	
High fat intake	1.30	0.92, 1.85	0.13	0.18
Low fat intake	2.01*	1.08, 3.74	0.03	
High protein intake	1.51*	1.00, 2.28	0.049	0.94
Low protein intake	1.48	0.93,2.35	0.10	

*CI* confidence interval, *GRS* genetic risk score, *OR* odds ratio

^a^Adjusted for gender and age

*p-value < 0.05

## Discussion

By using the GMM statistical method, we categorized the TPLS participants into three TMI-based growth trajectory groups: stable weight, decreased weight, and increased weight. After adjusting for gender, age, baseline TMI and other covariates, rs7206790 showed a significant association with the increased weight growth trajectory. Our study provided evidence for the association between the GRS of the four SNPs (namely rs7206790, rs2531995, rs4715210, and rs6548238) and the increased weight trajectory. Children with GRS ≥ 10 had a significantly increased risk to become obesity during adolescence compared with those with GRS < 10, and the influence seemed more remarkable for those with low calories intake.

Although many obesity-related GWAS have been conducted and have identified many possible genetic loci, relatively few studies have been designed to examine the genetic influence on the change of obese status in children. Through a systematic review of nine twin and five adopted children studies, Silventoinen et al. found that genetic factors had a considerably stronger influence on BMI variation than environmental factors did up to the age of 18 years [11]. Similar to the studies on adults, the strongest association was found in the SNPs of the *FTO* gene. More than 10 studies with different designs have explored the effects of genes on adolescence obesity since [40]. Warrington et al. have detected that the A allele at *FTO*/ rs1558902 was associated with the decrease in BMI in infancy, but showed an inverse effect from early childhood [41]. Besides, Graff et al. also found that the association between BMI and obesity-related loci, such as *FTO*, *TFAP2B*, and *TMEM18*, varied by age during adolescence and early adulthood, meaning that genetic consequences may change over time [42]. The genetic finding of this study was compatible with our GRS components, although the dominant SNPs may differ because of the change in ethnic groups.

Compared with normal body weight adolescents, individuals who are obese during pubertal growth have a significantly higher tendency to develop severe obesity in adulthood [43]. Moreover, our previous finding showed that a persistent increase in the TMI during adolescence is an independent risk factor for early-onset diabetes adjusting for childhood obesity status [9]. Obesity development in adolescents is not simply explained by energy balance disruption but may be a complex condition involving genetic, environmental, biological, and behavioral elements. Public obesity prevention strategies are mainly focused on the healthy promotion dimension, such as dietary habit modulation and encouraging physical activity. Because the increase in health awareness and lifestyle modification has been shown to be effective during the adolescent period when school-based interventions are applied [44] these approaches can be readily implemented during adolescence. Our finding that participants with a higher GRS would more easily develop obesity during adolescence, independent of baseline obesity status, is helpful for differentiating young children who are at a higher risk of obesity. Furthermore, the relationship between energy intake and obesity is more complex during adolescence, and our result supports the hypothesis that the genetic influence was more notable among the children with low dietary calories. Our research could help prevent the metabolic disorder problems resulting from obesity despite of low dietary caloriesby way of other lifestyle modification, should the genetic influence ring the alarm bell in the first place.

The main strength of our study is that anthropometric measurements were conducted every 3 months, and thus, we could easily capture different obesity development patterns during early adolescence and construct the growth trajectory. Moreover, we collected several possible confounding factors for the adolescent growth trajectory (including baseline obesity status, pubertal development, and environmental and behavioral factors) from the baseline questionnaire and physical examination, which enhances the accuracy of our results. Furthermore, few researchers have investigated the associations between obesity susceptibility variants and the change in obesity status across childhood. To our best knowledge, ours is the first study to investigate genetic influences on the TMI growth trajectory.

A limitation of our study is that most confounding factors were evaluated at baseline, and these conditions, especially exercise habits, may change and produce information bias. For example, Kwon et al. found that compared with consistently active participants, children who were active at first but decreased moderately to vigorous- intensity physical activity with age were more likely to become obese (OR: 2.77, 97% CI: 1.16–6.58) [13]. Academic performance is highly valued in Taiwanese high school students. In one study that assessed their physical activity, only 5.4% of Taiwanese adolescents (age: 13–15 years) met the World Health Organization recommendation for physical activity each day [45]. Because the overall physical activity performance of Taiwanese adolescents is suboptimal, the influence on the association between exercise habits and growth trajectory patterns may be tolerable. Besides, we used 24-hour dietary record to capture the nutrient intake of children. Even we have collected the information from 2 different days, it might be challenging to evaluate the long-term dietary consumption for adolescents without the follow-up longitudinal data. Moreover, the observation period of growth trajectory was not sufficiently long to distinguish more distinct growth trajectory groups. Nevertheless, the proportion of adolescents in the increased weight TMI growth trajectory group in this study was almost equal to the sum of children in the two different weight gain trajectory groups established using 6-year follow-up records in another independent cohort (9.7% vs. 9.3%) [9]. This result indicates that our TMI trajectory model was relatively stable during adolescence, and our GRS was a suitable tool for predicting children who were at a higher risk of obesity. Finally, we only recruited Taiwanese adolescents in the TPLS; whether the finding of our GRS could be applied to other ethnic populations needs to be confirmed in further studies.

In conclusion, *FTO* rs7206790 was significantly associated with an increase in the TMI during the adolescent period, and our 4-SNP GRS approach is a practical tool for determining cumulative genetic susceptibility and predicting the increased weight TMI growth trajectory, especially in the children with lower calories intake. However, further research with a larger sample size from a different population is necessary to confirm the gene–diet interaction as it concerns the adolescent growth trajectory.

## Data Availability

All of the data are available with reasonable request from the corresponding author.

## Abbreviations

BIC: Bayesian information criterion
BLRT: bootstrap likelihood ratio test
BMI: Body mass index
CI: confidence interval
GMM: growth mixture modeling
GRS: genetic risk score
GWAS: genome-wide association study
OR: odds ratio
SNP: Single-nucleotide polymorphism
TMI: tri-ponderal mass index
TPLS: Taiwan Puberty Longitudinal Study
